# Supplementary material for: Titanium(IV) Oxo-Complex with Acetylsalicylic Acid Ligand and Its Polymer Composites: Synthesis, Structure, Spectroscopic Characterization, and Photocatalytic Activity
Source: Materials (Basel). 2022 Jun 22;15(13):4408. doi: 10.3390/ma15134408 (PMC9267303; doi:10.3390/ma15134408)
Supplement: Supplementary file 1 [file materials-15-04408-s001.zip › materials-1754885-supplementary.pdf]

Table S1. Selected bond lengths [Å] and bond angles [°] of $[Ti_4O_4(O^iBu)_{10}(asp)_2]\cdot H_2O$ (**1**).

**Bond Lengths**			
Ti1-O71	1.785(2)	Ti3-O101	1.793(2)
Ti1-O6	1.840(2)	Ti3-O6	1.835(2)
Ti1-O61	1.971(2)	Ti3-O41	1.967(2)
Ti1-O31	2.014(2)	Ti3-O51	2.028(2)
Ti1-O5	2.0485(19)	Ti3-O5	2.053(2)
Ti1-O1	2.160(2)	Ti3-O12	2.158(2)
Ti2-O91	1.797(3)	Ti4-O121	1.787(2)
Ti2-O81	1.800(3)	Ti4-O111	1.821(2)
Ti2-O31	2.011(2)	Ti4-O51	2.008(2)
Ti2-O11	2.041(2)	Ti4-O2	2.039(2)
Ti2-O5	2.0561(19)	Ti4-O5	2.070(2)
Ti2-O41	2.104(2)	Ti4-O61	2.078(2)
Ti1-Ti3	2.9608(9)	Ti3-Ti4	3.1883(12)
Ti1-Ti4	3.1445(9)	Ti2-Ti3	3.1387(9)
Ti1-Ti2	3.1870(9)	Ti2-Ti4	3.9780(11)
Bond angles			
O(71)-Ti(1)-O(6)	100.39(10)	O(101)-Ti(3)-O(6)	99.51(10)
O(71)-Ti(1)-O(61)	102.87(10)	O(101)-Ti(3)-O(41)	102.95(10)
O(6)-Ti(1)-O(61)	93.51(10)	O(6)-Ti(3)-O(41)	93.48(9)
O(71)-Ti(1)-O(31)	99.89(10)	O(101)-Ti(3)-O(51)	99.13(10)
O(6)-Ti(1)-O(31)	98.33(10)	O(6)-Ti(3)-O(51)	99.65(10)
O(61)-Ti(1)-O(31)	151.94(9)	O(41)-Ti(3)-O(51)	152.02(9)
O(71)-Ti(1)-O(5)	176.65(10)	O(101)-Ti(3)-O(5)	176.07(10)
O(6)-Ti(1)-O(5)	80.13(9)	O(6)-Ti(3)-O(5)	80.12(9)
O(61)-Ti(1)-O(5)	80.37(8)	O(41)-Ti(3)-O(5)	80.98(8)
O(31)-Ti(1)-O(5)	76.76(8)	O(51)-Ti(3)-O(5)	77.12(8)
O(71)-Ti(1)-O(1)	94.11(10)	O(101)-Ti(3)-O(12)	93.90(10)
O(6)-Ti(1)-O(1)	165.19(9)	O(6)-Ti(3)-O(12)	166.14(9)
O(61)-Ti(1)-O(1)	80.26(9)	O(41)-Ti(3)-O(12)	80.04(8)
O(31)-Ti(1)-O(1)	81.96(9)	O(51)-Ti(3)-O(12)	81.52(9)
O(5)-Ti(1)-O(1)	85.56(8)	O(5)-Ti(3)-O(12)	86.74(8)
O(91)-Ti(2)-O(81)	97.45(14)	O(121)-Ti(4)-O(111)	96.64(12)
O(91)-Ti(2)-O(31)	98.17(10)	O(121)-Ti(4)-O(51)	100.68(11)
O(81)-Ti(2)-O(31)	97.44(10)	O(111)-Ti(4)-O(51)	97.55(10)
O(91)-Ti(2)-O(11)	91.20(10)	O(121)-Ti(4)-O(2)	87.87(10)
O(81)-Ti(2)-O(11)	97.50(10)	O(111)-Ti(4)-O(2)	96.69(10)
O(31)-Ti(2)-O(11)	161.16(8)	O(51)-Ti(4)-O(2)	162.40(9)
O(91)-Ti(2)-O(5)	95.55(11)	O(121)-Ti(4)-O(5)	96.38(10)
O(81)-Ti(2)-O(5)	166.39(12)	O(111)-Ti(4)-O(5)	166.67(10)
O(31)-Ti(2)-O(5)	76.65(8)	O(51)-Ti(4)-O(5)	77.18(8)
O(11)-Ti(2)-O(5)	86.24(8)	O(2)-Ti(4)-O(5)	86.66(8)
O(91)-Ti(2)-O(41)	171.09(10)	O(121)-Ti(4)-O(61)	168.90(10)
O(81)-Ti(2)-O(41)	89.72(12)	O(111)-Ti(4)-O(61)	90.17(11)
O(31)-Ti(2)-O(41)	86.09(9)	O(51)-Ti(4)-O(61)	87.05(9)
O(11)-Ti(2)-O(41)	82.57(8)	O(2)-Ti(4)-O(61)	82.62(9)
O(5)-Ti(2)-O(41)	77.75(8)	O(5)-Ti(4)-O(61)	77.43(8)
Ti(1)-O(5)-Ti(4)	99.56(8)	Ti(1)-O(5)-Ti(3)	92.41(8)
Ti(3)-O(5)-Ti(4)	101.30(8)	Ti(1)-O(5)-Ti(2)	101.87(8)

Ti(2)-O(5)-Ti(4)	149.23(10)	Ti(3)-O(5)-Ti(2)	99.60(9)
Ti(3)-O(6)-Ti(1)	107.33(11)		
Ti(3)-Ti(1)-Ti(4)	62.88(3)	Ti(1)-Ti(4)-Ti(3)	55.744(18)
Ti(3)-Ti(2)-Ti(1)	55.81(2)	Ti(1)-Ti(3)-Ti(2)	62.92(2)
Ti(3)-Ti(1)-Ti(2)	61.27(2)	Ti(1)-Ti(3)-Ti(4)	61.38(2)
Ti(4)-Ti(1)-Ti(2)	77.85(3)	Ti(2)-Ti(3)-Ti(4)	77.91(2)

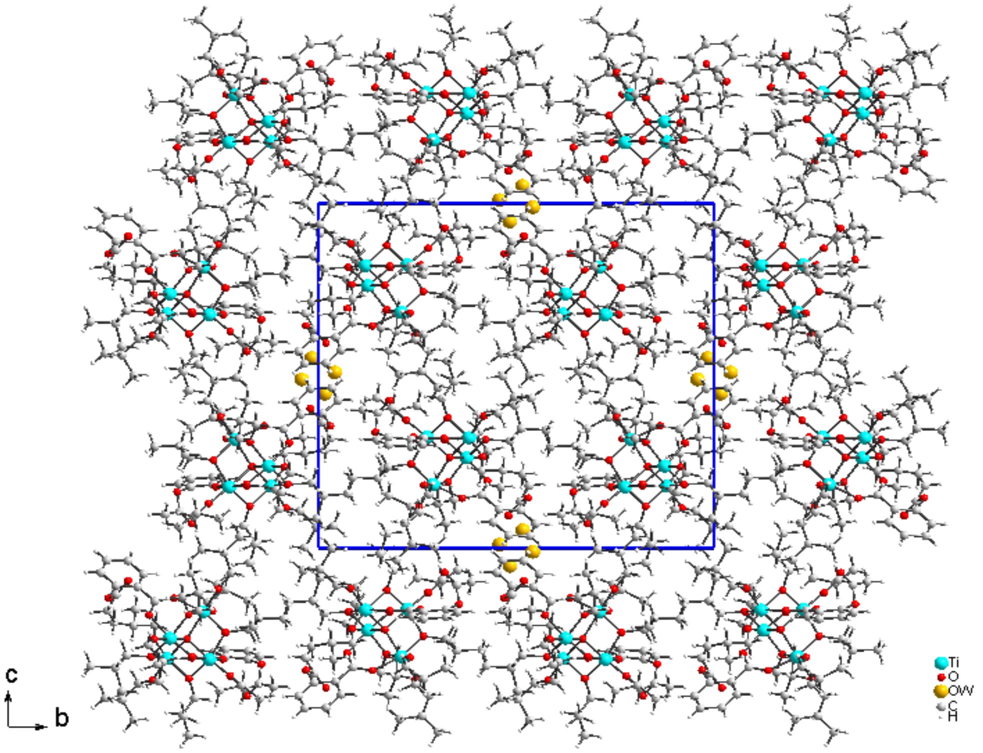

Figure S1. The crystal network of (**1**) along a axis shows densely packed titanium clusters. Water molecules (OW) are marked in orange and circled.

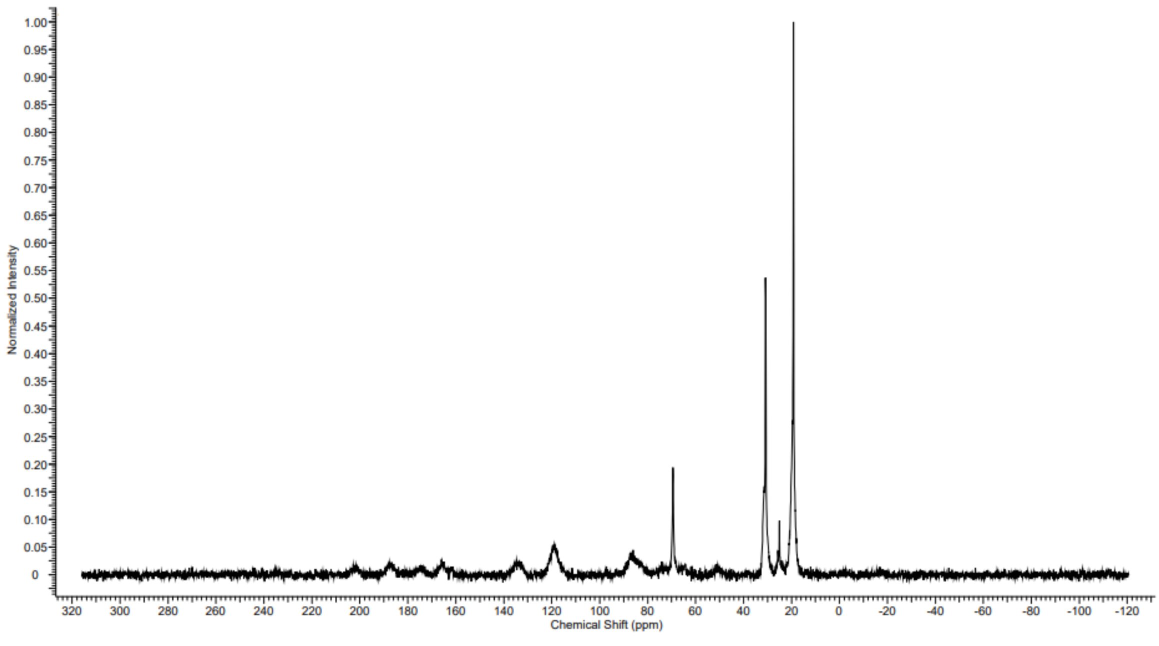

Figure S2. ^{13}C NMR spectrum of (**1**) in the solid phase.

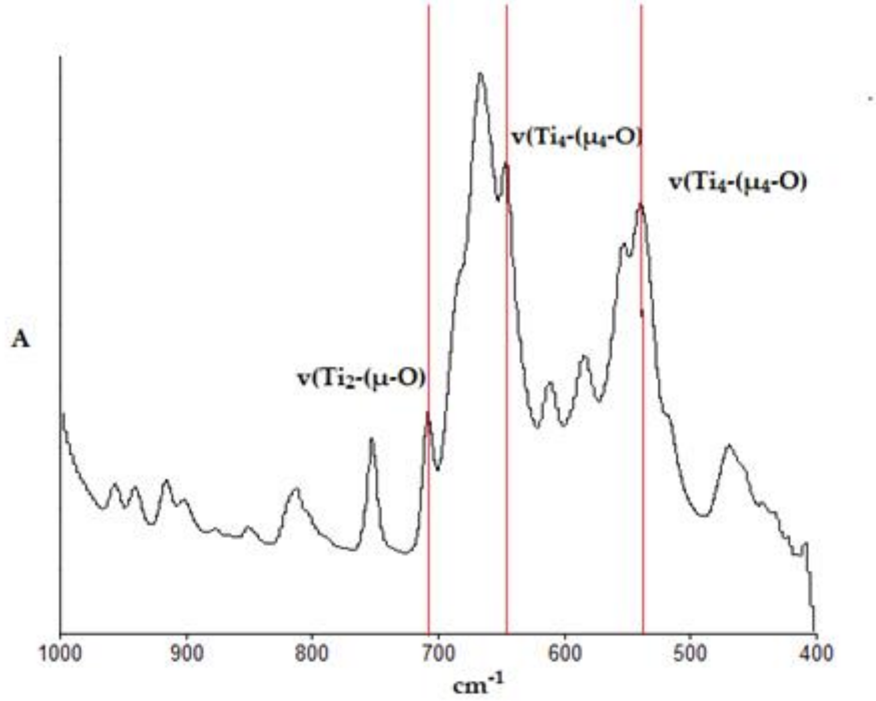

(**a**)

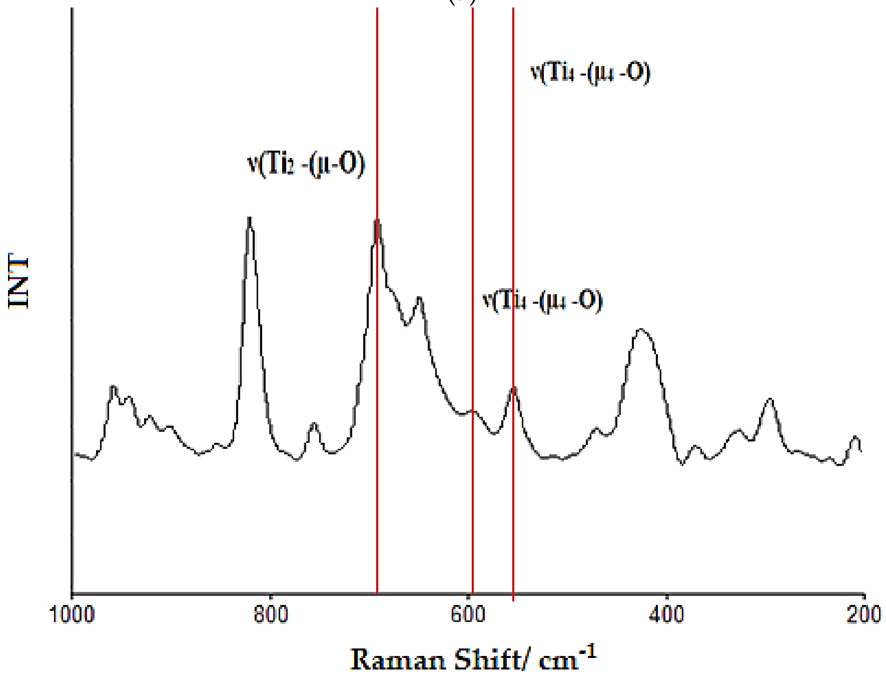

(**b**)

Figure S3. The IR (**a**) and Raman (**b**) spectra of the complex (**1**).

a)

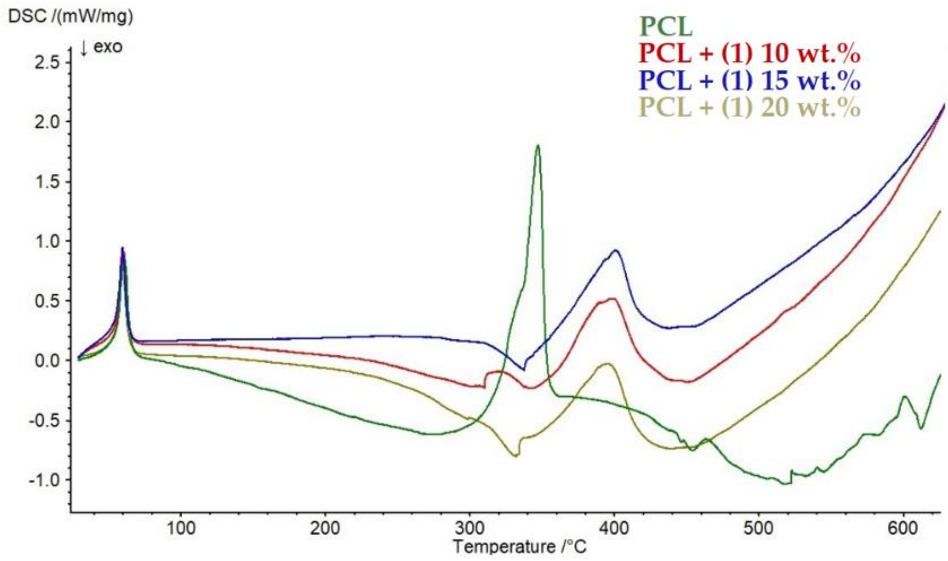

b)

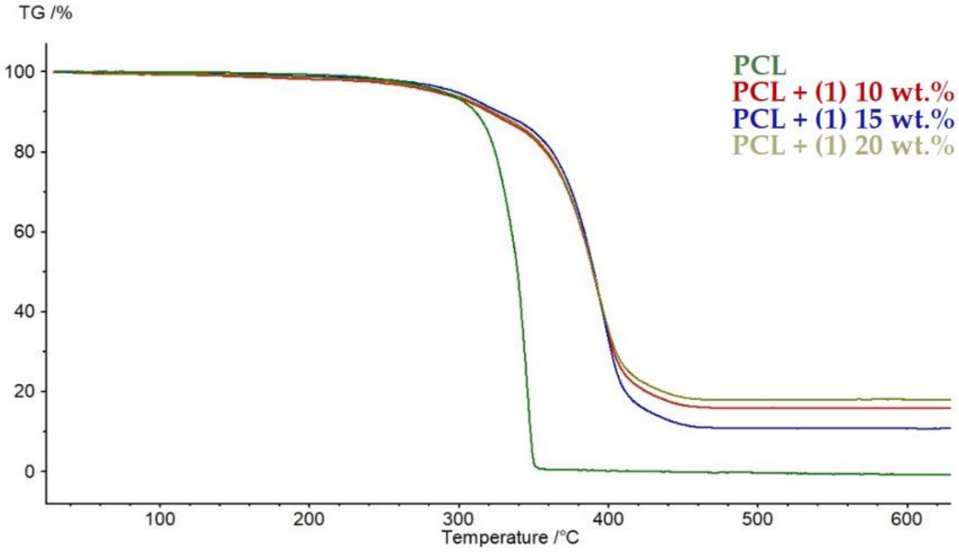

Figure S4. Differential scanning calorimetry curves (DSC) (**a**) and thermogravimetric curves (TG) and (**b**) of the produced composite materials and PCL.

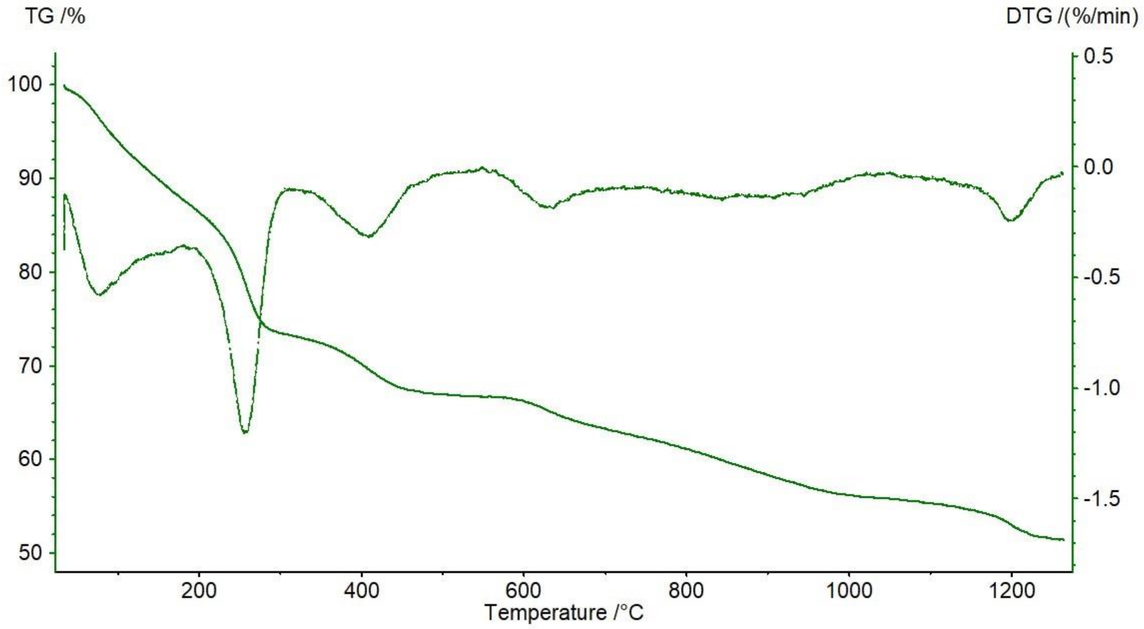

Figure S5. Thermogravimetric (TG) and derivative thermogravimetric (*DTG*) curves registered for (**1**) between 20 and 1300 °C.

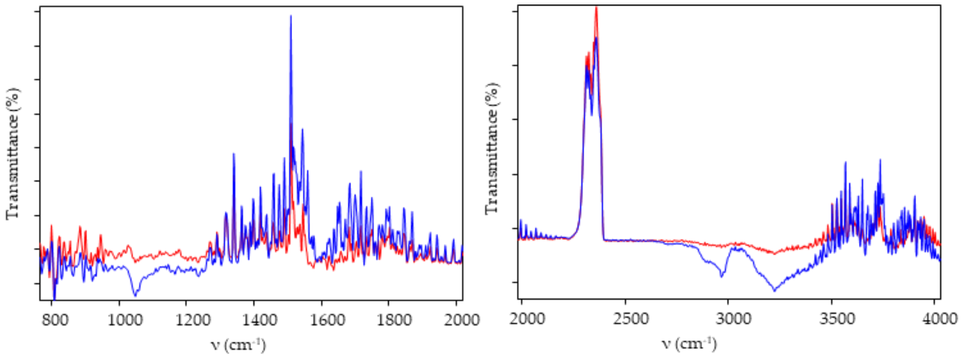

Figure S6. IR spectra of the volatile thermolysis products of (**1**), registered in the range 35–150 °C (red line—35 °C, blue line—150 °C).

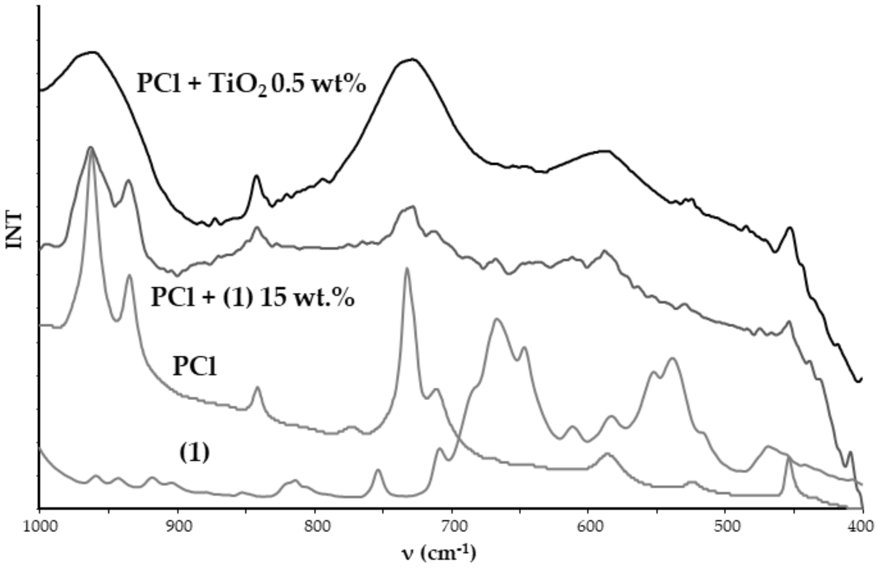

Figure S7. IR spectra of PCL + TiO_2 0.5 wt.%, PCL + (**1**) 15 wt.% samples, PCL, and (**1**).

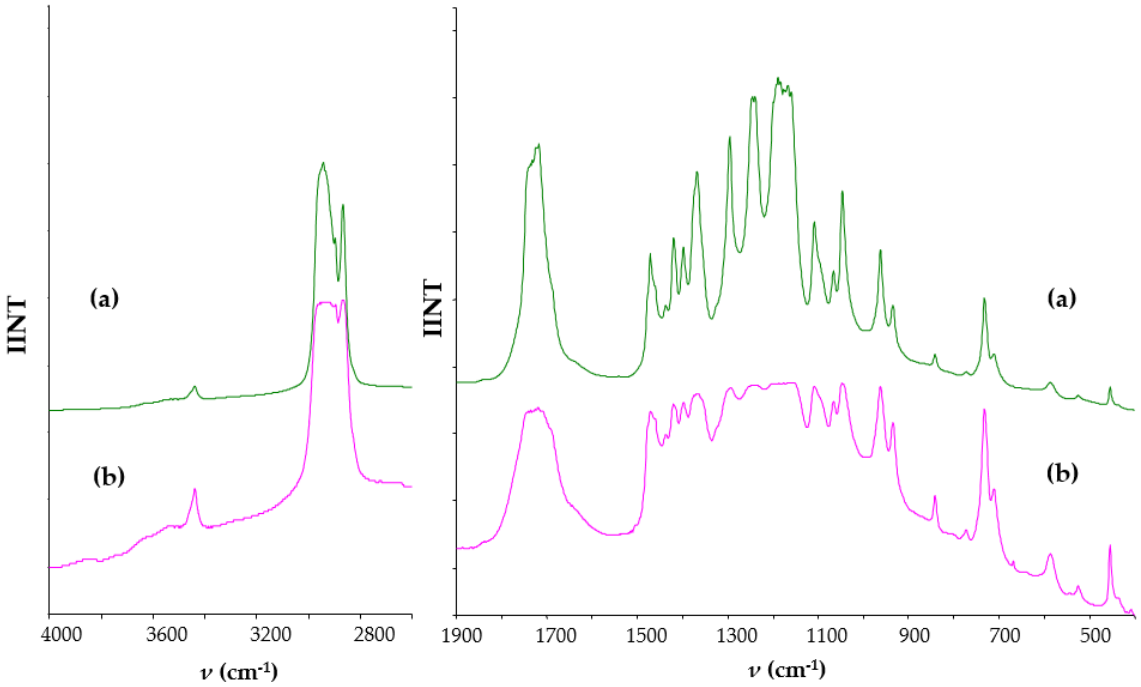

Figure S8. IR spectra of PCL before (**a**) and after (**b**) photocatalytic process. .
